# Trapped O_2_ and the origin of voltage fade in layered Li-rich cathodes

**DOI:** 10.1038/s41563-024-01833-z

**Published:** 2024-03-01

**Authors:** John-Joseph Marie, Robert A. House, Gregory J. Rees, Alex W. Robertson, Max Jenkins, Jun Chen, Stefano Agrestini, Mirian Garcia-Fernandez, Ke-Jin Zhou, Peter G. Bruce

**Affiliations:** 1 Department of Materials, University of Oxford, Oxford, UK; 2 The Faraday Institution, Didcot, UK; 3 Diamond Light Source, Didcot, UK; 4 Department of Chemistry, University of Oxford, Oxford, UK

**Keywords:** Batteries, Materials chemistry, Solid-state chemistry

## Abstract

Oxygen redox cathodes, such as Li_1.2_Ni_0.13_Co_0.13_Mn_0.54_O_2_, deliver higher energy densities than those based on transition metal redox alone. However, they commonly exhibit voltage fade, a gradually diminishing discharge voltage on extended cycling. Recent research has shown that, on the first charge, oxidation of O^2−^ ions forms O_2_ molecules trapped in nano-sized voids within the structure, which can be fully reduced to O^2−^ on the subsequent discharge. Here we show that the loss of O-redox capacity on cycling and therefore voltage fade arises from a combination of a reduction in the reversibility of the O^2−^/O_2_ redox process and O_2_ loss. The closed voids that trap O_2_ grow on cycling, rendering more of the trapped O_2_ electrochemically inactive. The size and density of voids leads to cracking of the particles and open voids at the surfaces, releasing O_2_. Our findings implicate the thermodynamic driving force to form O_2_ as the root cause of transition metal migration, void formation and consequently voltage fade in Li-rich cathodes.

## Abstract (web summary)

Oxygen redox cathodes deliver higher energy densities than those based on transition metal redox but commonly exhibit voltage fade on extended cycling. The loss of O-redox capacity and voltage fade is shown to arise from a reduction in O^2−^/O_2_ redox process reversibility and O_2_ loss.

## Main

Li-rich cathodes can deliver higher capacities than stoichiometric cathodes (up to 300 mAh g^−1^ versus ~220 mAh g^−1^ for LiNi_0.8_Co_0.1_Mn_0.1_O_2_), supported by the participation of both transition metal (TM) redox and O-redox^1–5^. However, the average voltage of the first discharge (~3.6 V) gradually diminishes and the load curve develops a step-like profile as the material is cycled. This ‘voltage fade’ phenomenon leads to a continuous loss of energy density over cycling, a disadvantage for commercialization of these materials.

One well-studied aspect of voltage fade is the gradual change in the redox reactions on the TMs. Over the first cycle, Co^3+/4+^ and Ni^2+/3+/4+^ are accepted to be the primary TM redox reactions in Li_1.2_Ni_0.13_Co_0.13_Mn_0.54_O_2_, with Mn remaining predominantly +4 (refs. ^6–9^). Over cycling, several studies have reported the increasing participation of low voltage Mn^3+/4+^ (refs. ^10–12^) and more recently Co^2+/3+^ (ref. ^13^). Such pronounced TM reduction is also more generally observed across a range of 3*d* and 4*d* cathodes and has been linked with irreversible out-of-plane TM migration and voltage fade^14–17^. While it appears that the increasing contribution of lower-voltage TM redox couples accompanies voltage fade, so far the underlying cause of voltage fade remains unclear.

A reduction in Li^+^ diffusivity on cycling, induced by, for example, structure disordering to form spinel or rocksalt-like surface layers^18–24^, could lead to a reduction in discharge voltage under the normal conditions of galvanostatic cycling between fixed voltage limits. However, such a reduction would be a kinetic overpotential. Galvanostatic intermittent titration technique measurements have shown that the voltage fade is predominantly not due to a larger overpotential, but is rather a thermodynamic voltage loss^25,26^. In terms of structural changes, previous studies have shown that the bulk structural reconfiguration is even more severe than the surface. The formation of nanopores has been identified in cycled materials by scanning transmission electron microscopy (STEM) and three-dimensional tomography^13,27^, and He pycnometry measurements have revealed a gradual decrease in the material density with cycling^28^. Chapman and co-workers also identified the growth of nanopores within the bulk on cycling with small-angle X-ray scattering (SAXS) measurements, but X-ray pair distribution function measurements could not determine whether they were filled or empty^29^. We showed recently, across a range of O-redox compounds, including Li_1.2_Ni_0.13_Co_0.13_Mn_0.54_O_2_, that O^2−^ oxidation results in the formation of molecular O_2_ that is primarily trapped in small voids in the bulk of these materials and can be reduced back to O^2−^ on discharge^30–32^. Following directly the fate of this O_2_ over cycling is critical to understanding the growth of nanopores and ultimately to explaining the origin of voltage fade.

In this Article, we follow directly and measure quantitatively the trapped O_2_ over cycling using high-resolution resonant inelastic X-ray scattering (RIXS) spectroscopy. We show that the amount of trapped O_2_ formed on charge gradually diminishes over cycling and that the trapped O_2_ that is formed is increasingly difficult to reduce back to O^2−^ on discharge. As the voids containing O_2_ grow, ^17^O nuclear magnetic resonance (NMR) data indicate thicker regions of insulating Li–O^2−^ form on the void surfaces, consistent with the O_2_ becoming increasingly difficult to reduce. ^129^Xe NMR and Brunauer–Emmett–Teller (BET) reveal increasing amounts of open voids at or near the surfaces over cycling, suggesting that as the voids grow large and the particle microstructure weakens, the particles crack releasing O_2_. Together, the accumulation of electrochemically inactive O_2_ in the particles and release of O_2_ from the opening of voids near the surface leads to reduction in the O-redox capacity. The loss of O^2−^/O_2_ redox capacity, which occurs primarily at potentials greater than 3 V, explains the observed voltage fade.

## Voltage fade characteristics in Li_1.2_Ni_0.13_Co_0.13_Mn_0.54_O_2_

**Fig. 1:**
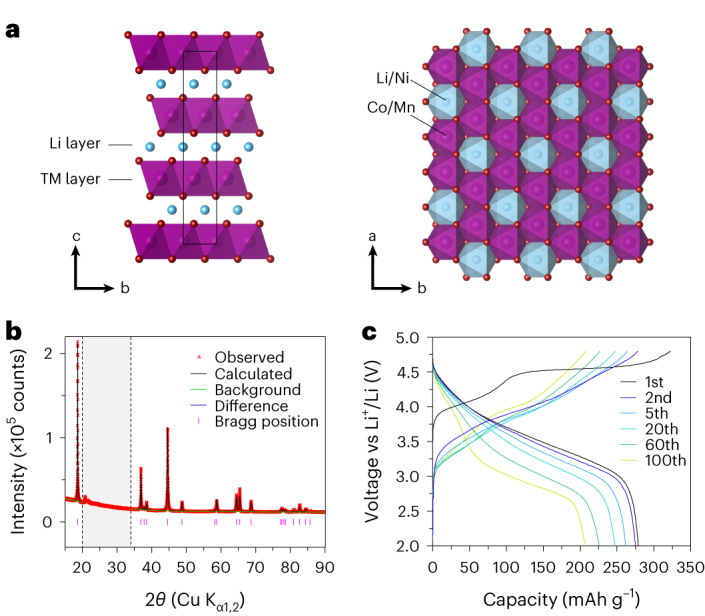
Structural characterization and electrochemical data for Li_1.2_Ni_0.13_Co_0.13_Mn_0.54_O_2_. **a**, Li_1.2_Ni_0.13_Co_0.13_Mn_0.54_O_2_ with a layered ${\rm{R}}\bar{3}{\rm{m}}$ structure, in-plane ordering of Li/Ni and Co/Mn giving rise to the honeycomb superstructure ordering. Li atoms are represented in blue, TM in purple and oxygen in red. **b**, PXRD data and refinement to the ${\rm{R}}\bar{3}{\rm{m}}$ crystal structure. **c**, Load curves for Li_1.2_Ni_0.13_Co_0.13_Mn_0.54_O_2_, cycled between 2.0 V and 4.8 V at 100 mA g^−1^ for 100 cycles.

**Extended Data Fig. 1:**
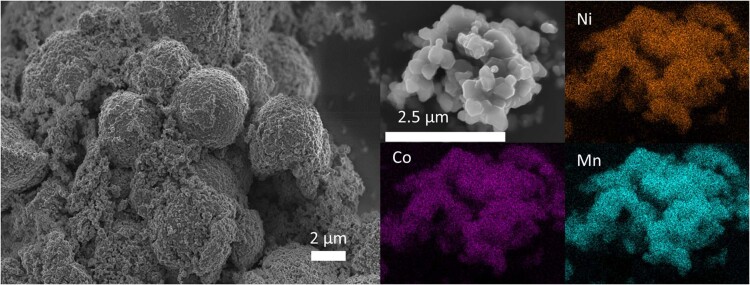
SEM and EDX images for Li_1.2_Ni_0.13_Co_0.13_Mn_0.54_O_2_showing elemental distribution. Spherical particles of 3–4 µm in diameter can be seen, while the EDX elemental analysis confirms a homogenous distribution of metals within the grains.

**Extended Data Fig. 2:**
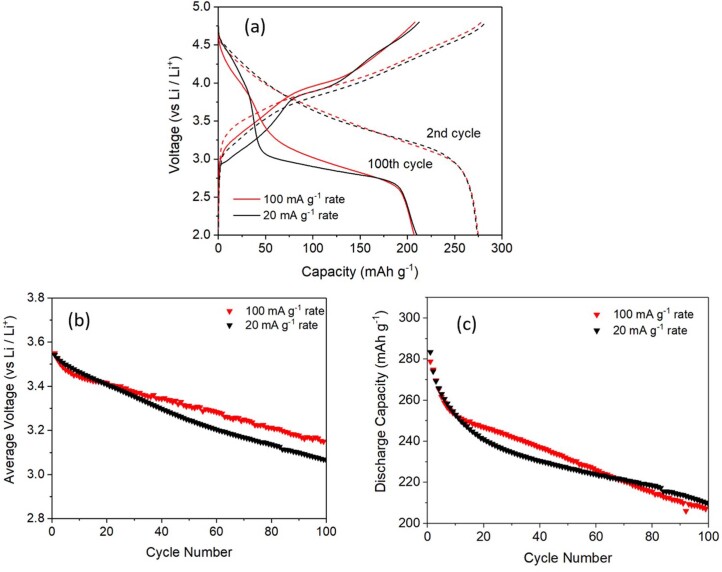
Electrochemical cycling data at different rates. (**a**) Cycling data collected at different current rates of 100 and 20 mA/g (C/3 and C/15). A similar degree of voltage (**b**) and capacity (**c**) fade is observed.

Samples of Li_1.2_Ni_0.13_Co_0.13_Mn_0.54_O_2_ were prepared by a co-precipitation synthesis (Methods). The composition was checked by inductively coupled plasma optical emission spectroscopy (Extended Data Table 1) and energy-dispersive X-ray spectroscopy (Extended Data Figs. 1 and 2), morphology by scanning electron microscopy (Extended Data Figs. 1 and 2) and structure by powder X-ray diffraction (PXRD; Fig. 1b and Extended Data Table 2). The structure of Li_1.2_Ni_0.13_Co_0.13_Mn_0.54_O_2_ involves O3-type stacking of the oxide layers, with a honeycomb arrangement of the TM and Li ions in the TM layer (Fig. 1a). The electrochemical load curves from the 1st to the 100th cycle are plotted in Fig. 1c. After the voltage plateau seen on the first charge, the load curve develops a continuous, sloping voltage profile. We have shown that this dramatic change is accompanied by the irreversible loss of honeycomb ordering to form vacancy clusters driven by the formation of molecular O_2_ (ref. ^31^). By the end of the first cycle, there is little evidence of the honeycomb superstructure peaks remaining in PXRD and negligible signal intensity to monitor over extended cycling. For this reason, the superstructure peaks were not included in our refinements here. From the 2nd to the 100th cycle, the load curve undergoes further changes, with a higher proportion of capacity at lower voltage on discharge, that is, voltage fade (Fig. 1c). This lowering of the average discharge voltage from the 2nd to the 100th cycle follows the same trend as previous reports of voltage fade on similar compounds^16,33^. A degree of capacity fade is also observed, similar to previously reported materials prepared in the same way^29,34–36^. Cycling was performed at a rate of 100 mA g^−1^ throughout the study. To confirm the voltage and capacity fade observed do not arise from kinetic limitations, cycling data were also collected at a lower rate of 20 mA g^−1^ over 100 cycles (Extended Data Fig. 2). These data show a very similar degree of voltage and capacity fade, confirming that these phenomena arise from bulk thermodynamic properties rather than kinetics.

## Redox changes on cycling

We showed previously, using high-resolution RIXS and ^17^O NMR, that molecular O_2_ is formed during O^2−^ oxidation in Li-rich cathodes^31^. To follow the changes in the amount of molecular O_2_ that is formed on cycling, we employed quantitative high-resolution RIXS at the O K edge. In the high resolution RIXS spectra, there are two main features associated with molecular O_2_: an energy loss feature at ~8 eV, and a series of vibrational progression peaks propagating from the elastic RIXS peak at 0 eV. To track the relative amount of O_2_, the area under the vibrational progression peaks (from 0.125 eV to 2.2 eV) was integrated. This feature was chosen as the peak intensity arises solely from molecular O_2_, with no contribution from the oxide ions. The analysis used to determine the amount of O_2_ over cycling is described in more detail in Methods.

**Fig. 2:**
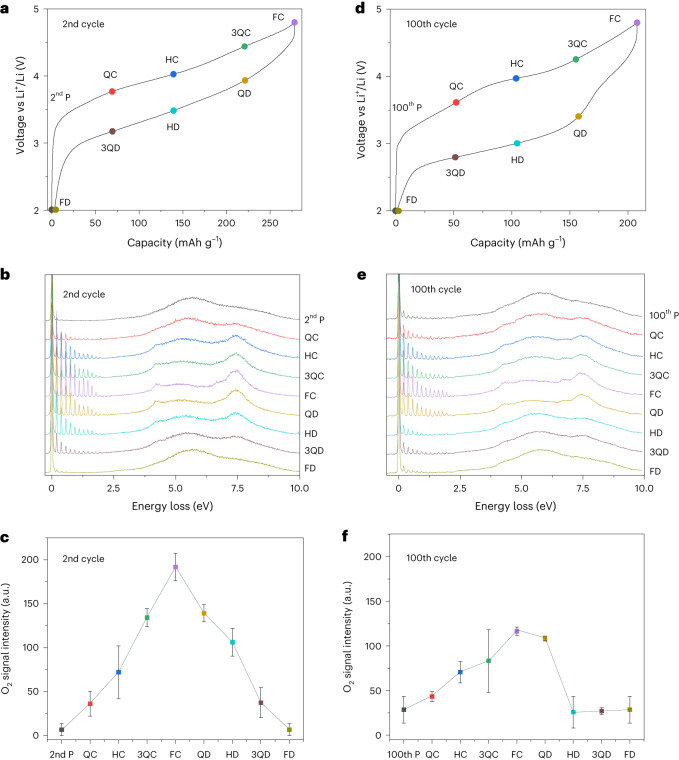
Evolution in bulk O-redox activity over 2nd and 100th cycles. **a**,**d**, Load curves for the 2nd (**a**) and 100th (**d**) cycles for Li_1.2_Mn_0.54_Co_0.13_Ni_0.13_O_2_, with the states of charge studied. **b**,**e**, RIXS spectra at 531.5 eV collected over the 2nd (**b**) and 100th (**e**) cycles. **c**,**f**, Variation in intensity of the molecular O_2_ signal in the RIXS spectra over the 2nd (**c**) and 100th (**f**) cycles, as determined by principal component analysis (Methods). Data are presented as mean ± standard deviation with a sample size of 15. P, pristine.

For this study, Li_1.2_Ni_0.13_Co_0.13_Mn_0.54_O_2_ was charged to specific points along the 2nd and 100th cycles, representing quarter charge (QC), half charge (HC), three-quarter charge (3QC), full charge (FC), quarter discharge (QD), half discharge (HD), three-quarter discharge (3QD) and full discharge (FD) (Fig. 2a,d). These points were defined on a fractional capacity basis of the total charge/discharge capacities of the 2nd and 100th cycles accordingly. At each state of charge, multiple RIXS scans were taken across different sample locations to minimize any effect of sample inhomogeneity, although we note there was little difference between the spectra (Extended Data Fig. 8). These scans were then averaged and plotted for both the 2nd and 100th cycles (Fig. 2b,e). The integrated signal intensity under the O_2_ vibrational progression peaks was plotted as a function of charge and discharge (Fig. 2c,f).

We observed substantial differences in oxygen activity over cycling. Throughout the charging process on the second cycle, the amount of O_2_ is seen to increase continuously over the full voltage range, which is mirrored by the decrease in O_2_ over the subsequent discharge (Fig. 2c). In contrast, while the 100th charge appears to show a continuous increase in O_2_, on discharge O_2_ reduction to O^2−^ occurs between the FD and HD points on the load curve. The HD point is at ~3 V, suggesting that the O^2−^/O_2_ redox couple is primarily active above 3 V on discharge, leaving the remainder of the discharge capacity to TM reduction. This accords with other studies reporting an increased contribution of lower-voltage Co^2+/3+^ and Mn^3+/4+^ redox couples after cycling^13,28^. While the majority of the O_2_ is observed to be reduced above 3 V, a small amount may be reduced at lower voltages.

**Fig. 3:**
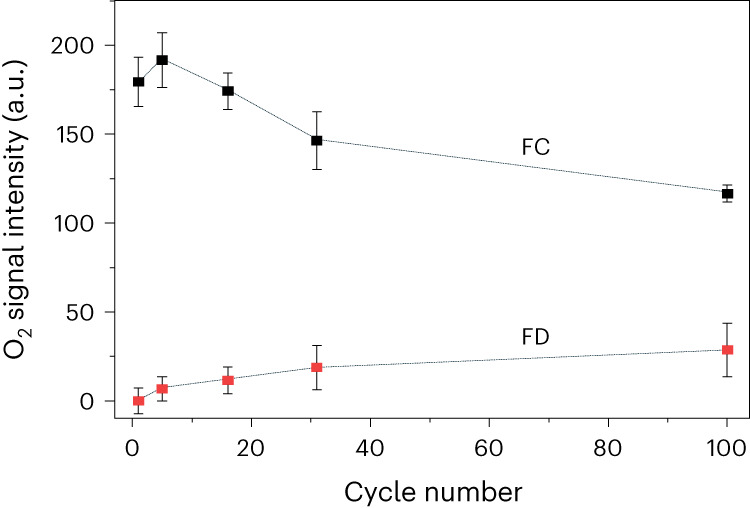
Evolution in amount of trapped O_2_ over cycling. Variation in intensity of the molecular O_2_ signal from RIXS over cycling in the fully charged (FC) and fully discharged (FD) states. The amount of O_2_ formed in the charged materials decreases with cycling and there is increasing evidence of O_2_ that is not reduced on discharge. Data are presented as mean ± standard deviation with a sample size of 15.

The intensity of the oxygen signal was tracked as a function of cycle number at FC and full discharge (Fig. 3). The data in Fig. 3 reveal an overall loss in the total amount of trapped O_2_ in the charged cathodes over cycling, with a 44% loss from the 2nd FC to the 100th FC. Furthermore, the amount of O_2_ remaining at the end of discharge appears to increase from the 2nd to the 100th cycle (Fig. 3). The accumulation of O_2_ at the end of discharge signals that the trapped O_2_ is becoming increasingly electrochemically inactive. Taking the difference between the O_2_ at the end of charge and discharge reveals that the amount of O-redox capacity diminishes from 0.48 e^−^ per formula unit on the 2nd cycle to 0.22 e^−^ per formula unit on the 100th cycle. As a percentage of the total charge passed O-redox diminishes from 55% on the 2nd cycle to 34% on the 100th cycle, the balance of the capacity being made up by TM redox. The loss of O-redox capacity on cycling arises in part from the formation of electrochemically inactive O_2_ that is still trapped but cannot be reduced on each cycle but also from the overall loss of O_2_ from the particles, reflected in the 44% loss of O_2_ at the end of charge after 100 cycles (Fig. 3). The remaining active O-redox capacity on the 100th cycle (0.22 e^−^ per formula unit) aligns with the charge passed between FC and HD, that is, the charge passed above 3 V. Overall the loss of O-redox activity, through a combination of electrochemically inactive O_2_ and release of O_2_ from the particles, can account for the reduced contribution of charge above 3 V, leading to voltage fade and much of the capacity loss on cycling Li_1.2_Ni_0.13_Co_0.13_Mn_0.54_O_2_.

## Void formation on cycling

**Fig. 4:**
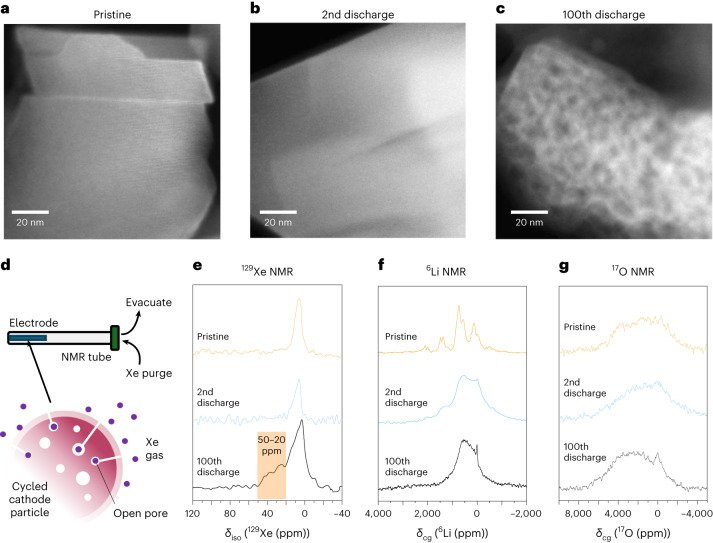
Formation of voids and large diamagnetic Li-rich regions over cycling. **a**–**c**, ADF-STEM images showing single grains of the pristine (**a**), 2nd discharge (**b**) and 100th discharge (**c**) material showing the formation of voids about 4–12 nm in diameter over extended cycling. **d**, ^129^Xe NMR experiments. Samples were extracted from cells and infiltrated with Xe gas to probe the open porosity. **e**, ^129^Xe NMR of the pristine, 2nd discharge and 100th discharge materials. The orange region highlighted indicates the presence of open voids of 17 nm diameter and greater after 100 cycles. *δ*_iso_, isotropic chemical shift. **f**,**g**, ^6^Li (**f**) and ^17^O (**g**) NMR isolating slow and fast relaxing environments. The sharp peaks at 0 ppm in the ^6^Li and slow relaxing ^17^O NMR spectra indicate the formation of large diamagnetic Li-rich regions on extended cycling.

**Extended Data Fig. 4:**
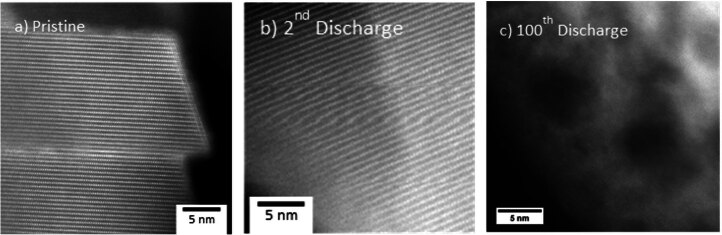
Additional ADF-STEM images. (**a**) Pristine, (**b**) 2^nd^ cycle and (**c**) 100^th^ cycle. Again, well defined atomic layers can be seen within the grains in the pristine and 2^nd^ cycles, while the images from the 100^th^ cycle highlight the presence of voids.

**Extended Data Fig. 5:**
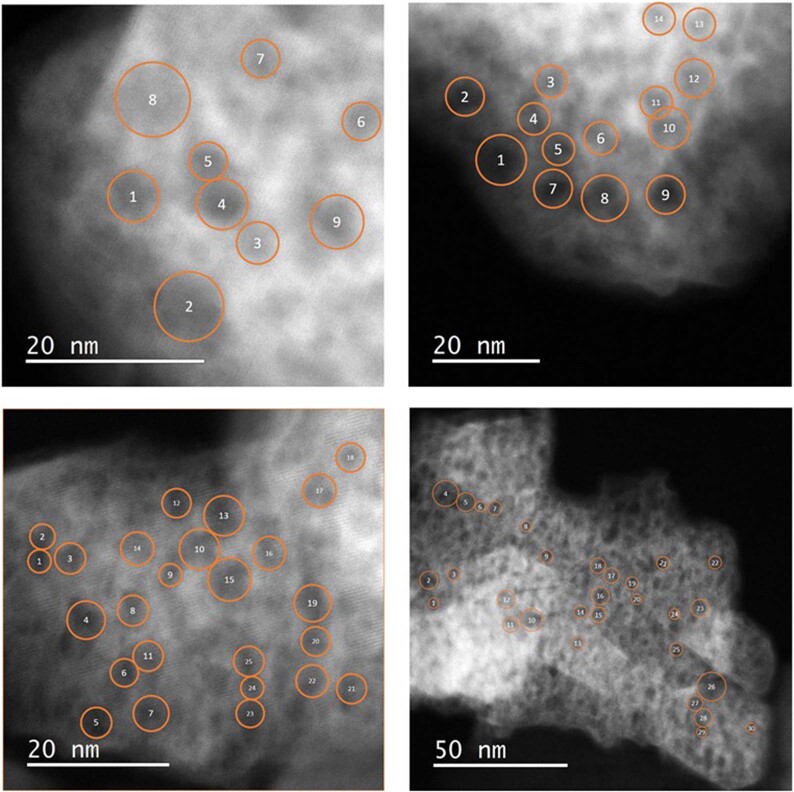
ADF-STEM images for samples collected after the 100^th^ cycle. Voids are outlined in orange and range in dimensions from 4 to 12 nm. The likely interconnection of several of these voids is apparent.

To examine the origin of the diminishing extent of O_2_ formation and reduction with cycling, annular dark field (ADF)-STEM imaging was carried out to probe changes in the particle microstructure. The images shown in Fig. 4a–c and Extended Data Figs. 4 and 5 illustrate substantial changes within individual grains of Li_1.2_Ni_0.13_Co_0.13_Mn_0.54_O_2_ after 100 cycles, consistent with previous reports identifying void formation and growth using SAXS, STEM and three-dimensional tomography^13,27–29^. Comparing the images for the pristine and 2nd discharge with the 100th discharge clearly shows the development of very extensive voiding with a high density of voids, as seen by the darker areas. The voids vary from about 4–12 nm in size and appear to be distributed throughout the particle. Given the high density of voids it is likely that a number of these voids are interconnected leading to pores of larger dimensions than is apparent from STEM.

**Extended Data Fig. 6:**
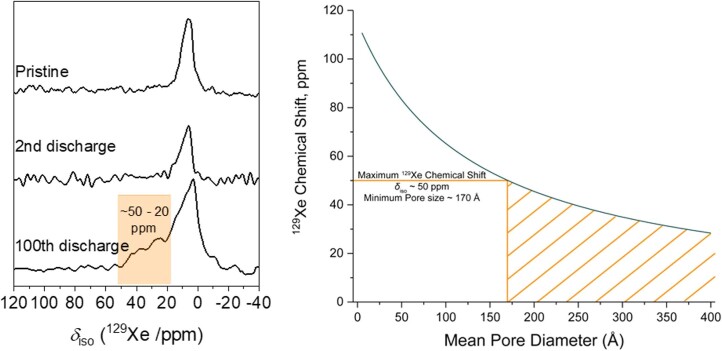
^129^Xe NMR data. ^129^Xe NMR data at different stages of cycling alongside a plot of void size vs chemical shift^45^. The minimum void size in the samples is around ~17 nm, as taken from a maximum chemical shift of 50 ppm.

**Extended Data Fig. 7:**
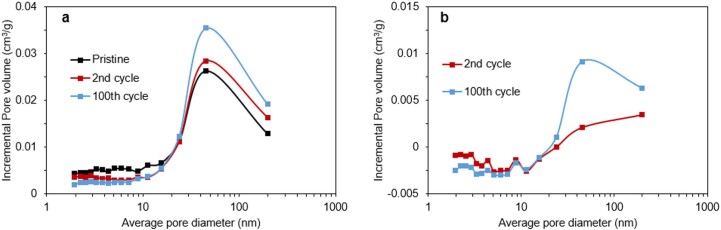
BET data. (**a**) BET data showing the pore size distributions for the pristine, 2^nd^ cycle and 100^th^ cycle samples. To remove the contribution from carbon and binder which were present in all samples, the pristine data were subtracted from the 2^nd^ and 100^th^ cycle data to better observe the changes in cathode particle porosity, (**b**).

To investigate the extent to which the voids are closed or open, ^129^Xe NMR was used. Xenon-129 (*I* = 1/2) is an inert gas with a large polarizable electron cloud, giving xenon a wide chemical shift range^37^. When ^129^Xe is constrained within a void, it comes into contact with the surface of the void causing the electron cloud to be distorted and a chemical shift to be observed^38^. This shift has a well-known relationship with void size that has been experimentally determined, described in Extended Data Fig. 6 (ref. ^39^). Li_1.2_Ni_0.13_Co_0.13_Mn_0.54_O_2_ was first degassed under dynamic high vacuum for 48 h to evacuate the sample, before being flushed with xenon gas (Fig. 4d). The xenon spectra for samples of the pristine, 2nd and 100th discharge material in Fig. 4e all show resonances centred at ~10 ppm, consistent with bulk xenon gas being paramagnetically broadened due to interactions with the surface of the cathode material. The signal could arise from large (>40 nm) open pores at the surface, although no such pores are evident in the ADF-STEM. After 100 cycles, increased spectral density between 20 ppm and 50 ppm is observed in the ^129^Xe NMR spectra. This signal is not present in the pristine or second cycled material, meaning new open voids with a minimum size of 17 nm are forming (Fig. 4e). This new signal would not arise if O_2_ had been lost directly from the surface, only Xe atoms in a partially confined pore open to the surface experience a chemical shift and so there must be increased surface porosity. These NMR observations are supported by BET measurements which also show there is an increase in the number of open pores at the surface, >20 nm in diameter between the 2nd and 100th cycles (Extended Data Fig. 7). The somewhat larger pore sizes from NMR and BET are consistent with some of the pores seen in STEM being interconnected and hence larger than the STEM images suggest.

## The contents of the closed voids

In addition to the ^129^Xe NMR, ^6^Li and ^17^O solid-state magic angle spinning (MAS) NMR were used to investigate changes over cycling. The ^6^Li solid-state MAS NMR of the pristine material (Fig. 4f) shows two regions of resonances; the resonance that arises from Li in the TM layer is at ~1,500 ppm and is consistent with well-defined honeycomb ordering, and those between 400 ppm and 900 ppm are from Li in the alkali metal layers^31,40^. After two cycles, broadening of the signal and substantial shift in the centre of gravity of the resonance (from ~700 to ~350 ppm) is observed, consistent with local disordering of the cathode and clustering of Li into more diamagnetic regions with a reduced number of TM neighbours, these changes have been discussed previously^31^. After 100 cycles, a notable sharp resonance at 0 ppm is observed that is consistent with Li in an extended diamagnetic environment.

The corresponding ^17^O MAS NMR spectra (Fig. 4g) of the pristine material show a broad amorphous line shape with very limited resolution. This is attributed to a range of oxide-ion environments that are broadened due to paramagnetic interactions. After 100 cycles at the end of discharge, the spectral density of the resonance has shifted to a slightly higher frequency, suggesting TM-rich regions are forming, that is, where the oxide ions are coordinated by several TM ions (O–TM_3_, O–TM_4_…). There is also evidence of the formation of a peak at ~0 ppm. These O atoms exhibit a similar diamagnetic chemical shift in the ^17^O NMR spectrum to lithia, Li_2_O. As trapped O_2_ is reduced back to O^2−^, the Li^+^ ions reinserted into the particles surround the O^2−^ resulting in the nanoscopic regions of Li_2_O (refs. ^31,41^). This is consistent with the 100th cycle ^6^Li spectrum and can be attributed to the formation of Li-rich regions in the cathode (O–Li_5_ and O–Li_6_). Together, the ^17^O and ^6^Li NMR suggests that the materials segregates into regions of highly paramagnetic TM-rich and diamagnetic Li-rich clusters over extended cycling.

**Fig. 5:**
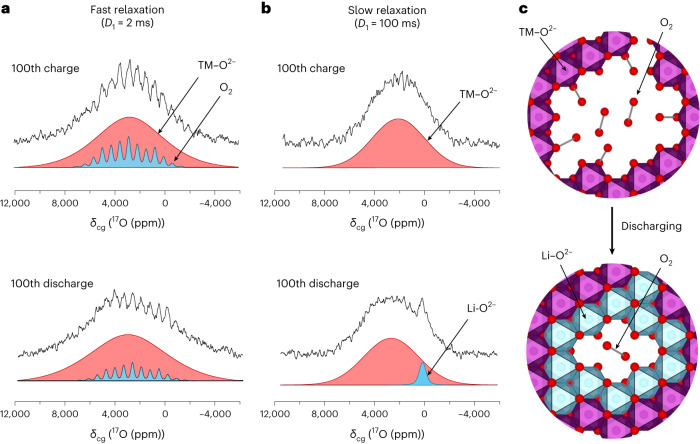
Partial reduction of O_2_ trapped in voids to form Li-coordinated O^2−^ on the 100th discharge. **a**,**b**, ^17^O NMR spectra isolating fast (**a**) and slow (**b**) relaxing ^17^O environments. The sharp peaks in **a** are assigned to trapped molecular O_2_, which decrease in intensity on discharge. There is still evidence of some residual molecular O_2_ in the discharged sample, *δ*_cg_(^17^O_2_) = 2,770 ppm. In **b** the slow relaxation ^17^O is dominated by oxide environments coordinated to paramagnetic TM ions (TM–O^2−^), *δ*_cg_ = 2,100–2,300 ppm. After discharge, a new ^17^O environment is formed corresponding to oxide surrounded by Li (that is, Li–O^2−^) created by the reduction of O_2_ in the voids and reinsertion of Li^+^ into the voids coordinated by the O^2−^, centred at *δ*_cg_ = 0 ppm. *D*_1_, relaxation delay. **c**, Large voids accommodating O_2_ are partially repopulated by Li^+^ on discharge. Most O_2_ is reduced to O^2−^ but some residual O_2_ remains.

To probe for molecular O_2_ over the 100th cycle using ^17^O NMR, fast relaxing spectra (2 ms), compared with those discussed above (100 ms), were collected to selectively enhance the oxygens which experience substantial paramagnetic relaxation enhancement. O_2_ molecules, possessing two unpaired electrons, are expected to have a much stronger paramagnetic relaxation enhancement than oxides. The data (Fig. 5) show a well-defined chemical environment centred at *δ*_cg_ = 2,770 ppm, where *δ*_cg_ is the chemical shift centre of gravity, with a manifold of spinning sidebands, consistent with previous measurements of trapped molecular O_2_ (refs. ^31,42^). A decrease in the intensity of the O_2_ resonance can be seen between the 100th charge and discharge as the O_2_ is reduced to O^2−^; however, there is evidence of some residual O_2_ present in the 100th discharged sample. These results are in accord with our RIXS measurements (Figs. 2 and 3) and indicate that O_2_ is only partially reduced over the 100th cycle. The corresponding slow relaxing spectra (Fig. 5b) reveal that the ^17^O diamagnetic peak arising from O^2−^ surrounded predominantly by Li, Li–O^2−^, is completely absent in the 100th charge sample. This evidence supports the conclusion that the closed voids seen in ADF-STEM are filled with O_2_ and that upon discharge this oxygen is partially reincorporated into the lattice as O^2−^ in ionic Li-rich regions (Fig. 5c), accompanied by the reinsertion of Li^+^ ions into the void space coordinated by the O^2−^, as we have described previously^31^.

## O_2_ loss and residual trapped O_2_ explain voltage fade

We showed recently, using high-resolution RIXS and ^17^O NMR, that on the first cycle of the O-redox Li_1.2_Ni_0.13_Co_0.13_Mn_0.54_O_2_ material, O^2−^ is oxidized to O_2_ with the loss of honeycomb TM ordering and formation of small vacancy clusters trapping the O_2_ molecules distributed throughout the particle. The formation of trapped O_2_ occurs quickly on charging as evidenced by the lack of electron-hole states on the O^2−^ sublattice. By the end of the first cycle, the trapped O_2_ is completely reduced back to O^2−^, accounting for the reversible O-redox capacity.

The results presented here show that, on subsequent cycling, the O-redox mechanism is not static and continues to evolve, although more gradually. On cycling, there is increasing accumulation of O_2_ at the end of discharge (Fig. 3), indicating that not all O_2_ formed on charge is reduced on the subsequent discharge, that is, there is a decrease in the reversibility of the O^2−^/O_2_ transformation. The decrease in the amount of O_2_ trapped at the top of charge also signals a loss of O_2_ from the particles. Together, the loss of O_2_ and the decreasing reversibility of the O^2−^/O_2_ transformation lead to a loss of 0.26 e^−^ per formula unit in O-redox capacity, corresponding to a reduction in the percentage of the capacity due to O-redox from 55% on the 2nd cycle to 34% on the 100th cycle. As the O^2−^/O_2_ couple occurs above 3 V, the loss of O-redox capacity at the expense of a higher proportion of TM capacity leads to the overall voltage loss on cycling. The decrease in the reversible O-redox capacity is also commensurate with the capacity fading on cycling, that is, the capacity loss on cycling is associated with reduction in the O-redox activity at the higher voltages and a lower average voltage.

**Fig. 6:**
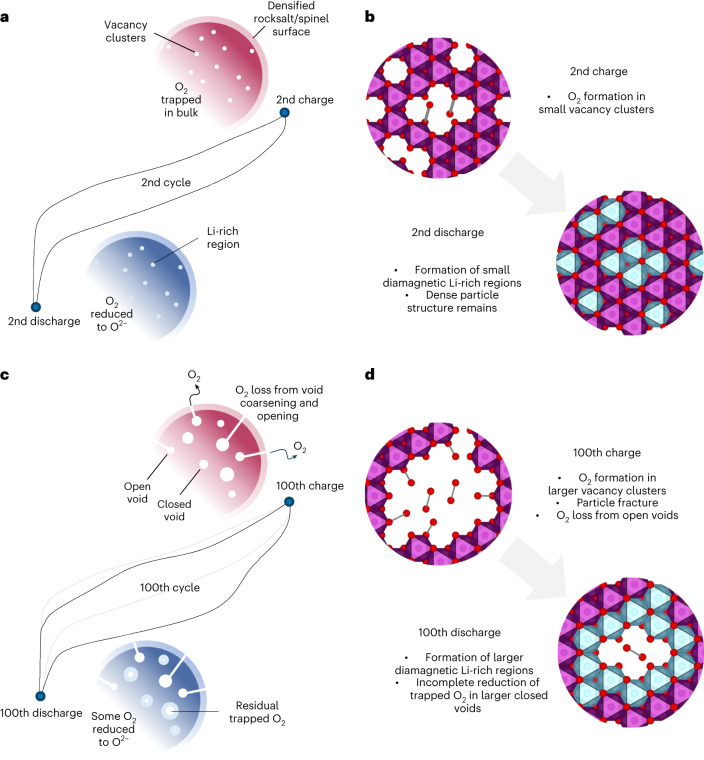
Voltage fade mechanism. **a**,**b**, Second cycle: reversible O-redox involves the formation of molecular O_2_ trapped in small vacancy clusters throughout the particle. O_2_ molecules are fully reduced to O^2−^ on discharge forming small diamagnetic Li-rich regions. **c**,**d**, One-hundreth cycle: further TM migration leads to agglomeration and coarsening of clusters into larger voids driven by the formation of more O_2_. The large voids and their high density in the particles lead to a weakening of the latter, cracking and O_2_ release. It is also more difficult to reduce O_2_ in larger voids.

Accompanying the loss of O-redox capacity, Li-rich materials exhibit pronounced changes in the cathode particle microstructure. There have already been a number of reports of voids forming on cycling in Li_1.2_Ni_0.13_Co_0.13_Mn_0.54_O_2_ using STEM, ptychography and small-angle scattering^27–29^. Voids also manifest as a reduction in average particle density, which has been recently observed^28^. Our ADF-STEM images (Fig. 4a–c and Extended Data Figs. 4 and 5) provide additional evidence for this, showing voids develop that are about 4–12 nm in diameter within individual particles after 100 cycles. Previous studies proposed void formation on the first charge corresponding to a few vacant cation sites and therefore approximately 1 nm in diameter, implying the voids grow on cycling. Reduction of O_2_ trapped in these larger voids is expected to be more difficult than in the much smaller voids present on the first and second cycles. This is in accord with the RIXS observations at the end of discharge showing increasing amounts of unreduced O_2_ over cycling and by ^17^O NMR, which also shows evidence of residual O_2_ at the end of the 100th discharge. Furthermore, our ^17^O NMR study of the 100th cycle discharge process reveals that, as the trapped O_2_ is reduced to O^2−^, diamagnetic ^17^O environments form that were not present in the charged sample, indicative of O^2−^ in ionic, Li-rich environments. This evidence further supports the conclusion that the closed voids formed on cycling are filled with O_2_ and that on discharge some of the O_2_ is reduced to O^2−^, which is reincorporated into the lattice along with the charge compensating reinserted Li^+^ (Fig. 6). The insulating nature of these Li–O^2−^ regions that will form on the walls of the void where the electrons to reduce O_2_ on discharge are supplied offers an explanation for why it is increasingly difficult to reduce O_2_ in larger voids.

The ^129^Xe NMR and BET data show that the cathode particles develop increased density of open voids >17 nm in diameter at the particle surfaces on cycling. There is also a wider body of evidence showing that Li-rich NMC suffers from particle cracking, which had commonly been associated with increased lattice strain^43^. Together this suggests that the increased density of relatively large voids filled with O_2_ upon cycling may result in weakening and hence fracturing of the particles, releasing O_2_ from open or partially open voids at or near the surfaces of the particles and explaining its loss on extended cycling release (Fig. 6).

## Implications

Suppressing the release of O_2_ from particles by protecting the surface with coatings is known to be an effective strategy to prevent capacity fade in Li-rich cathodes and it can also suppress voltage fade to an extent. However, a key implication of our study is that surface coatings cannot eliminate voltage fade. Efforts must be directed towards bulk mitigation strategies such as avoiding O_2_ formation and the appearance of voids in favour of stabilised hole states on O (ref. ^32^).

The oxygen redox process, which proceeds by the formation and reduction of trapped O_2_ molecules, becomes less prevalent on cycling Li_1.2_Ni_0.13_Co_0.13_Mn_0.54_O_2_. On charging, the O_2_ formed is trapped in closed voids within the particles. The trapped O_2_ becomes increasingly electrochemically inactive because the growth in size of these closed voids makes electron tunnelling between the O_2_ and the void edges more difficult. The voids at or near the surface, including any new fracture surfaces due to particle cracking, are open and can vent O_2_. Together these two mechanisms result in the loss of O-redox capacity on cycling. The gradual loss of O_2_ participating in the charge compensation reaction over extended cycling offers an explanation for the voltage fade phenomenon which draws together the observations of structural reorganisation, void formation, void opening and TM reduction into a single mechanism. The implication is that voltage fade mitigation strategies should focus on the bulk and suppressing the formation of O_2_.

## Methods

### Co-precipitation synthesis

Ni_0.13_Mn_0.54_Co_0.13_ CO_3_ precursors were prepared by a coprecipitation route. NiNO_3_·6H_2_O (≥98%, Sigma-Aldrich), MnNO_3_·4H_2_O (≥99%, Sigma-Aldrich) and CoNO_3_·6H_2_O (≥99%, Sigma-Aldrich) were dissolved in de-ionized water with a molar ratio of 0.13:0.54:0.13 to prepare a 1.5 M solution. In addition, a 1.5 M solution of Na_2_CO_3_ (≥99.5%, ACS reagent, Sigma-Aldrich) was prepared. The solutions were added dropwise into a beaker under continuous stirring, at a constant temperature of 40 °C and pH 7.6. After full addition of the TM solution, the beaker was left covered overnight under stirring. The resulting carbonate mix was then washed with de-ionized water, filtered and dried at 120 °C overnight. The dried mixed metal carbonate precursor was then mixed with Li_2_CO_3_ (≥99%, ACS reagent, Sigma-Aldrich) using a mole ratio of (Li:TM of 1:1.5) and calcined at 900 °C for 15 h under continuous O_2_ flow to obtain the desired compound. A heating and cooling rate of 5 °C min^−1^ was used during the synthesis. ^17^O-labelled samples were prepared in the same way except the final calcination step was performed under a sealed atmosphere of O_2_ gas (CortecNet >70 atom% ^17^O).

### Electrochemical characterization

The electrodes were prepared by combining the active material (80 wt%), Super P carbon (10 wt%) and polytetrafluoroethylene binder (10 wt%) using a mortar and pestle. The mixture was then rolled to a thickness of about 100 μm to form self-supporting films. Electrodes were assembled into coin cells using Whatman glass fibre separators and 1 M LiPF_6_ in ethylene carbonate:dimethyl carbonate 50:50 (battery grade, Sigma-Aldrich) electrolyte, with a Li metal counter electrode. A typical coin cell has an active mass loading of ~10 mg. Galvanostatic cycle testing was carried out using Maccor Series 4000. The cells and electrodes were prepared and assembled/disassembled in the glove box under an inert atmosphere and all cycling for the characterization studies was performed using the same conditions. Cells were cycled between 2.0 V and 4.8 V versus Li^+^/Li at a rate of 100 mA g^−1^ without voltage holds, rests or formation cycling.

### Inductively coupled plasma optical emission spectroscopy

The pristine cathode material was dissolved in aqua regia (HCl:HNO_3_/25:75), before diluting the solution for measurement. A calibration curve was created using standard solutions. Elemental analysis was carried out by ion-coupled plasma optical emission spectroscopy using a PerkinElmer Optima 7300DV ion-coupled plasma optical emission spectroscope.

### PXRD

Diffraction data were collected on a Rigaku 9 kW SmartLab Cu-source diffractometer equipped with a Hypix 2D detector.

### ADF-STEM

ADF-STEM micrographs were measured using an aberration-corrected JEOL ARM 200F microscope operated at 200 kV. A convergence semi-angle of 22 mrad was used, with a collection semi-angle of 69.6–164.8 mrad (ADF). Sets of fast-acquisition multiframe images were taken and corrected for drift and scan distortions using SmartAlign43. To avoid the exposure to air, sample transfer to the STEM microscope was carried out with a vacuum transfer suitcase.

### RIXS

**Extended Data Fig. 8:**
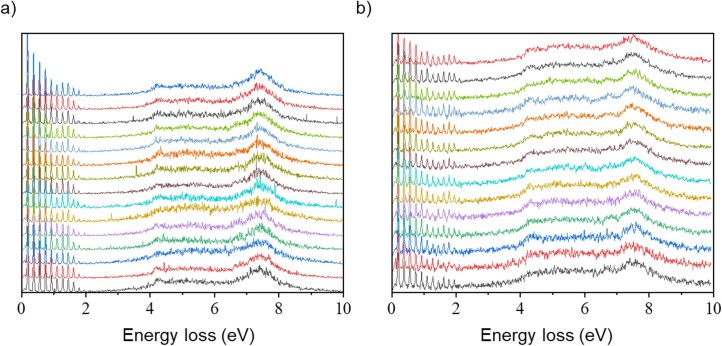
RIXS line scans. RIXS line scans collected at 531.5 eV at different sample locations for the charged samples of (**a**) 2^nd^ and (**b**) 100^th^ cycles.

High-resolution RIXS data were collected using the I21 beamline at Diamond Light Source^44^. To produce the data sets for the quantitative analysis, scans at 531.5 eV were recorded at 15 different sample locations and averaged together, with little inhomogeneity in the signal observed (Extended Data Fig. 8). The line scan data from 0.1 eV up to 13.0 eV, excluding the signal from the elastic peak, were *z*-scored by dividing each scan by its standard deviation. Then, the area under the vibrational peak progression (from 0.13 eV to 2.2 eV) was integrated to measure the relative amount of O_2_. The areas for each scan were averaged to create a measure of oxygen intensity, with errors coming from the standard deviation of the mean for each data set.

### Solid-state ^17^O and ^6^Li MAS NMR spectroscopy

All ^6^Li and ^17^O MAS (*υ*_R_ = 37037 Hz) solid-state NMR were completed at 9.45 T (*υ*_0_^6^Li = 58.92 MHz, *υ*_0_^17^O = 54.25 MHz) using a Bruker Avance III HD spectrometer and a 1.9 mm double air bearing MAS probe, where *υ*_R_ is the MAS frequency and *υ*_0_ is the Larmor frequency. All ^6^Li and ^17^O spectra are referenced to 1 M ^6^LiCl_(aq)_ and H_2_^17^O, respectively, at 0 ppm. All spectra were recorded using a Hahnecho (^*π*^/_2_–*τ*–*π*–*τ*) sequence, where *τ* is 1/*υ*_R_ and ^*π*^/_2_ is 250 kHz; the resultant free induction decay is processed as a half echo. The ^6^Li spectra were achieved with a recycle delay of 300 ms. These spectra were completed with relaxation times of 2 ms (fast relaxation) and 100 ms (slow relaxation).

### ^129^Xe static NMR

A J-Young NMR tube containing the Li-rich NMC cathode, was degassed under dynamic high vacuum using a turbo pump for 48 h and then infilled with natural abundance xenon gas (BOC) at 1 atm of pressure for 48 h. The ^129^Xe NMR (298.1 K, 1 atm) spectrum were completed at 9.45 T (*υ*_0_ = 110.69 MHz) using a 5 mm solution-state NMR probe at a controlled temperature of 298.1 K. A 25 kHz pulse was utilized for all experiments with a recycle delay of 0.5 s. All shifts are referenced to natural abundance Xe (gas, 1 atm and 298.1 K) at 0 ppm.

### BET

Nitrogen adsorption/desorption analysis was carried using a Micromeritics 3Flex Adsorption Analyser. Samples were dried via in situ degassing at 70 °C for 5 h before measurement.

## Online content

Any methods, additional references, Nature Portfolio reporting summaries, source data, extended data, supplementary information, acknowledgements, peer review information; details of author contributions and competing interests; and statements of data and code availability are available at 10.1038/s41563-024-01833-z.

## Figures and Tables

**Extended Data Fig. 3:**
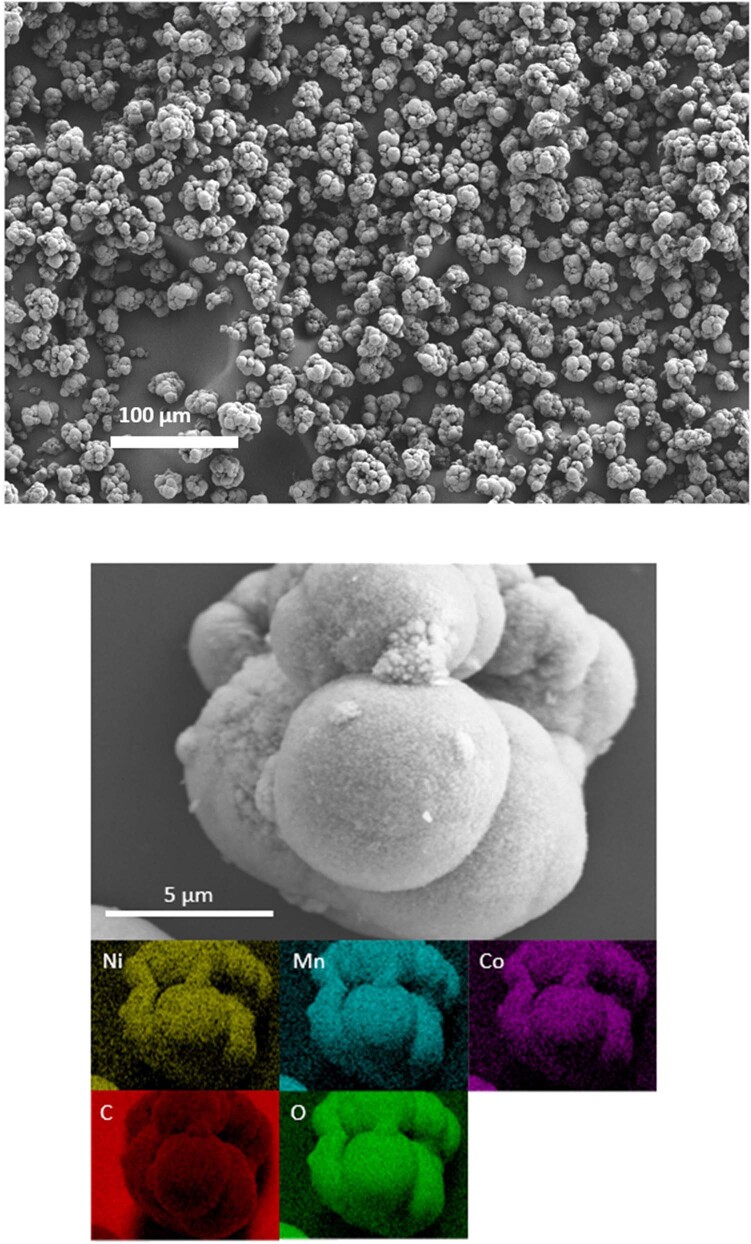
SEM and EDX for the mixed metal carbonate precursor. Spherical particles of 3–4 µm in diameter can be seen in the SEM and EDX shows a homogenous distribution of metals within grains and between particles.

**Extended Data Table 1:**
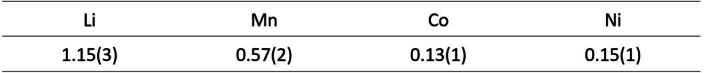
ICP-OES data for Li_1.2_Ni_0.13_Co_0.13_Mn_0.54_O_2_

**Extended Data Table 2:**
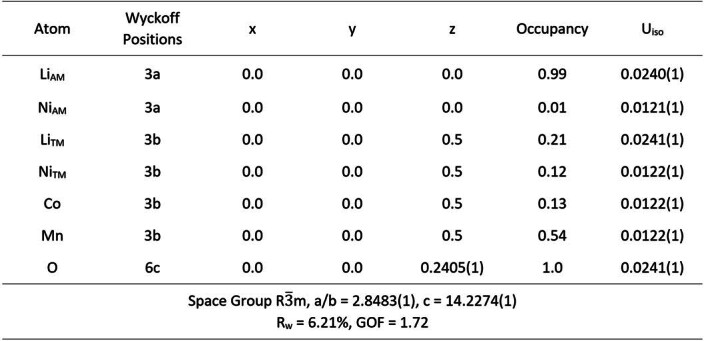
Rietveld Refinement parameters of powder X-ray diffraction data for Li_1.2_Ni_0.13_Co_0.13_Mn_0.54_O_2_

## Data Availability

All the data generated or analysed during this study are included within the paper and its Extended Data figures and tables. Source data are available from the corresponding authors upon reasonable request.
